# Supplementary figures and images for: Noteworthy trends in maladaptive coping strategies and hindrances to help-seeking behaviour among adolescents living in Malaysia’s People’s Housing Project (PPR) during the COVID-19 pandemic: A qualitative study
Source: PLoS One. 2025 Mar 21;20(3):e0318381. doi: 10.1371/journal.pone.0318381 (PMC11927882; doi:10.1371/journal.pone.0318381)

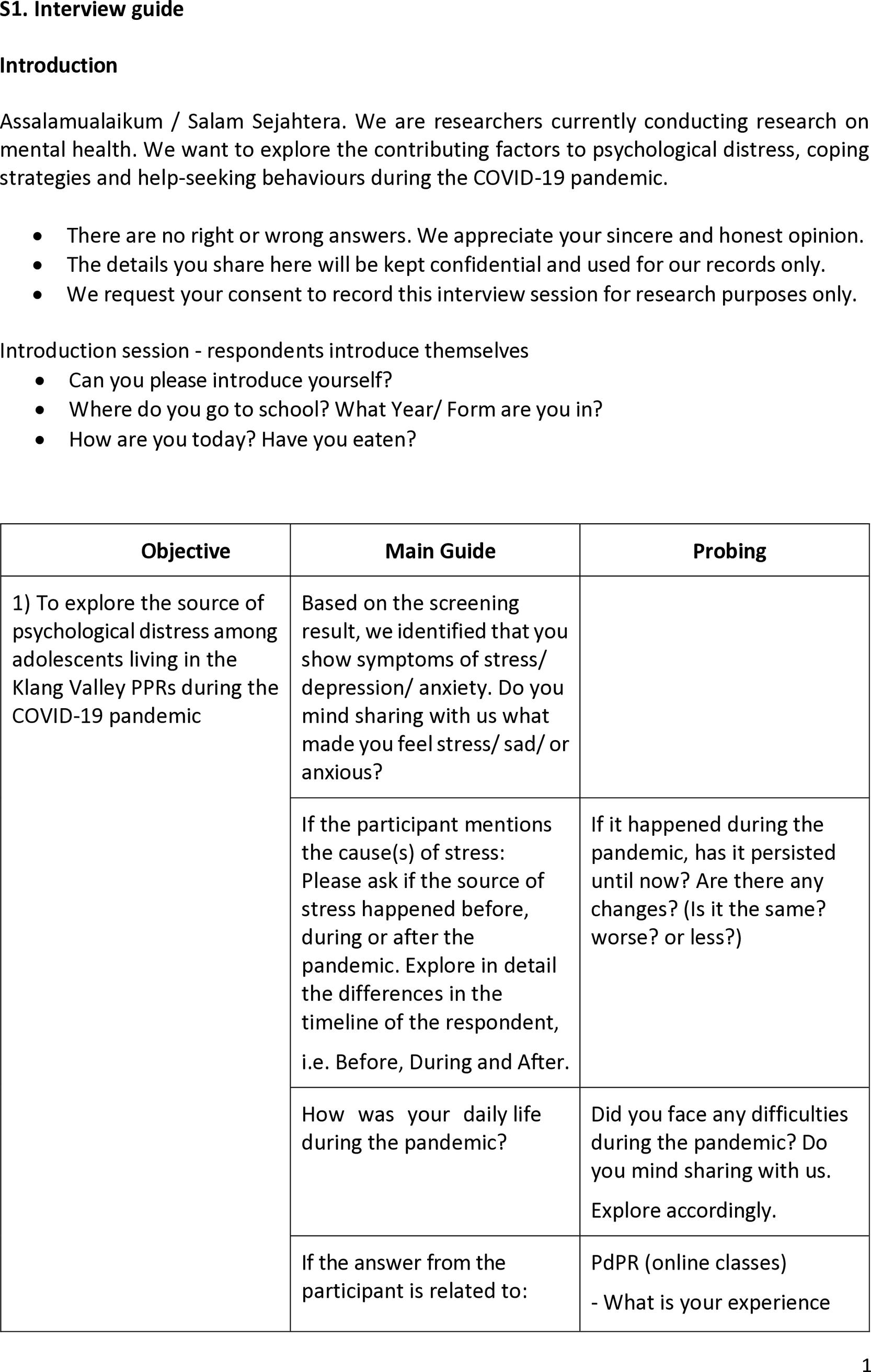

Supplement: S1 File — (ZIP) [file pone.0318381.s001.zip › S1_File 1.tif]

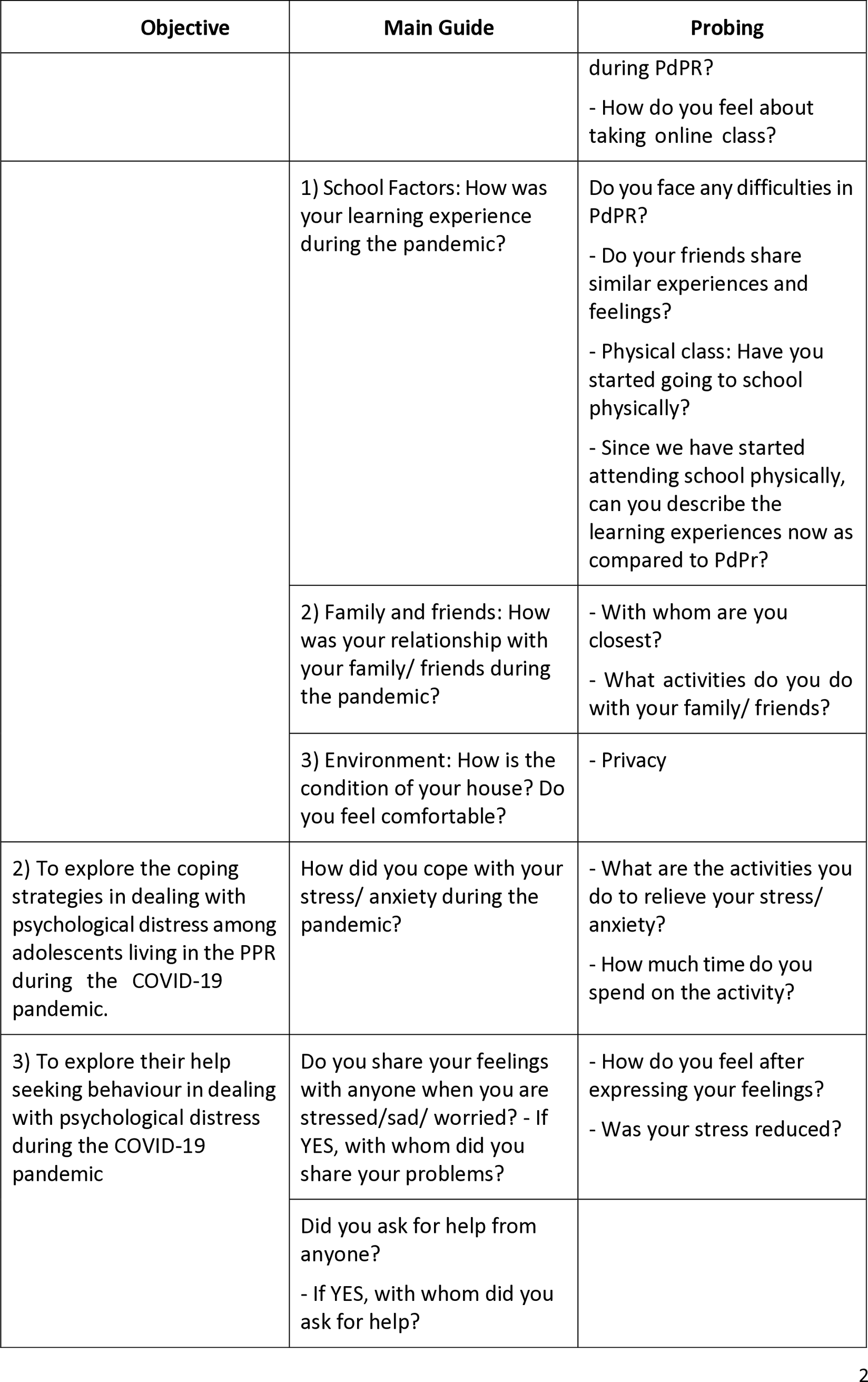

Supplement: S1 File — (ZIP) [file pone.0318381.s001.zip › S1_File2.tif]

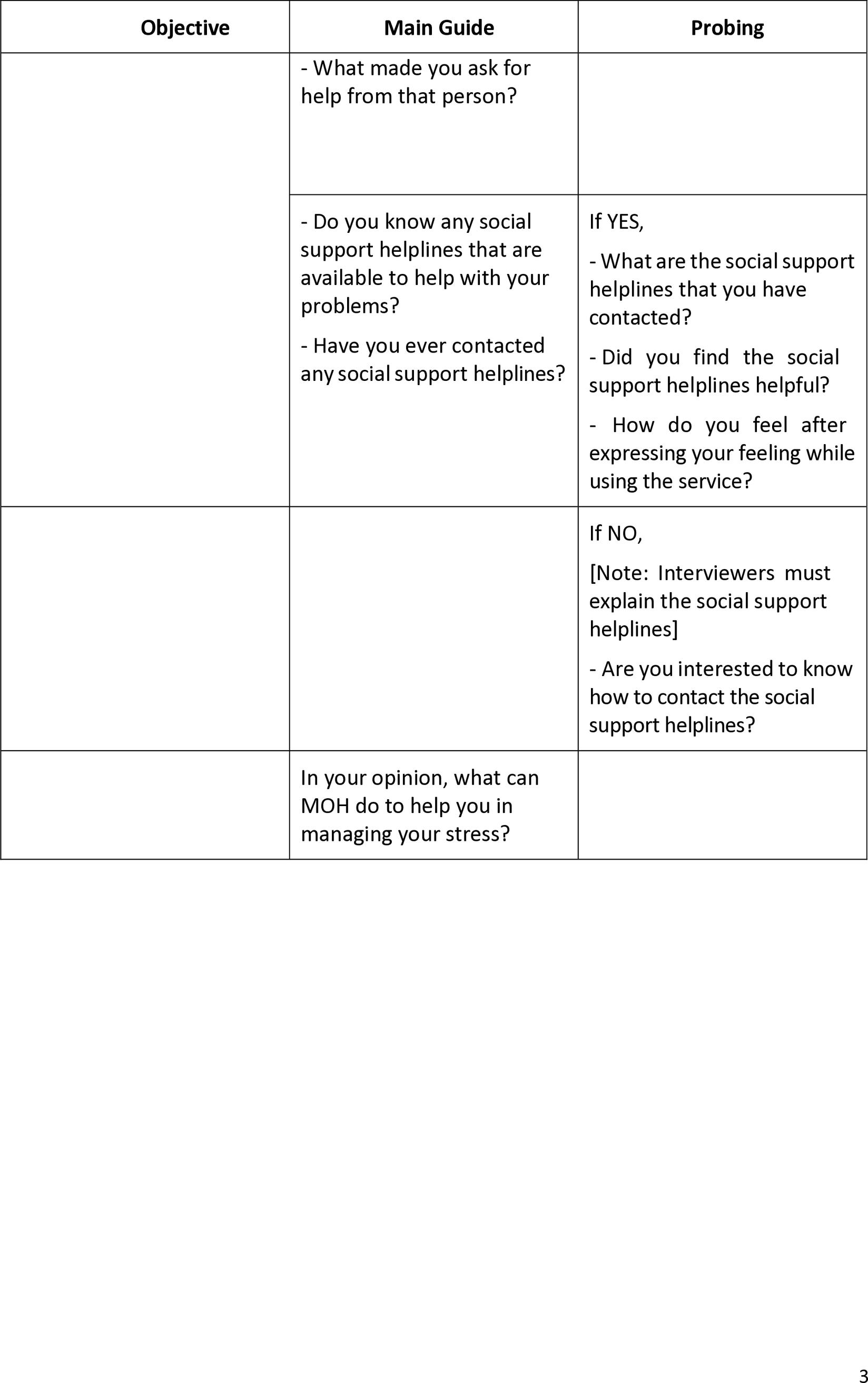

Supplement: S1 File — (ZIP) [file pone.0318381.s001.zip › S1_File3.tif]
