# Supplementary material for: Noteworthy trends in maladaptive coping strategies and hindrances to help-seeking behaviour among adolescents living in Malaysia’s People’s Housing Project (PPR) during the COVID-19 pandemic: A qualitative study
Source: PLoS One. 2025 Mar 21;20(3):e0318381. doi: 10.1371/journal.pone.0318381 (PMC11927882; doi:10.1371/journal.pone.0318381)
Supplement: Attachment — Submitted filename: Reviewer report on maladaptive coping strategies.pdf [file pone.0318381.s003.pdf]

Reviewer report on noteworthy trends in maladaptive coping strategies and hindrances to help-seeking behaviour among adolescents living in Malaysia's People's Housing Project (PPR) during the COVID-19 pandemic: qualitative study

# 1. Abstract

1. **Clarity and Structure**: The abstract could benefit from clearer wording, particularly in describing the research question and the study's significance. Opening with a concise statement on the specific challenges faced by adolescents in Malaysia's People's Housing Project (PPR) communities due to the COVID-19 pandemic would improve focus.
2. **Relevance of the Research Question**: Adding a sentence on why this study is particularly relevant to PPR communities in Malaysia would strengthen the justification and make the abstract more compelling.
3. **Methods**: Consider adding a phrase on the sample size (e.g., number of participants) and the specific methods used (e.g., semi-structured interviews), as this provides readers with a clearer understanding of the study's scope.
4. **Results** :To improve clarity, specify some of the maladaptive coping strategies and barriers identified (e.g., avoidance, lack of trust in mental health services). Including one or two specific examples would make the findings more tangible for the reader.
5. **Conclusions and Implications:** Strengthen the conclusion by mentioning potential implications for policy or practice. For example, a brief statement about how this research could inform mental health interventions tailored to disadvantaged communities would make the abstract more impactful.

# 2. Introduction

- To strengthen the relevance, consider providing a brief overview of the unique characteristics of the PPR communities (e.g., socio-economic constraints, limited access to mental health resources). This would contextualize the challenges faced by adolescents in these settings and reinforce the importance of the study.
- While the authors have referenced international studies on adolescent coping and help-seeking, the literature review could benefit from further discussion of any regional or cultural factors specific to Southeast Asia or Malaysia, which might influence coping strategies and help-seeking behaviors. This would provide additional context for the study's relevance
- Consider explicitly stating the research question or hypothesis at the end of the introduction. This would give readers a clear understanding of the study's focus and objectives.

# 3. Methods

- A more detailed explanation of the coding and analysis process would improve transparency. Specifically, it would be beneficial to describe how coding was

conducted (e.g., use of software, independent coding by multiple researchers) and how disagreements were resolved to ensure inter-coder reliability.
- Additionally, mentioning whether the researchers used any form of reflexivity to minimize bias would strengthen the methodological robustness.

## 4. Results
- Including some quantitative data, such as the proportion of participants endorsing each coping strategy or barrier, would provide additional context and help quantify the prevalence of specific behaviors.
- Furthermore, a summary table outlining each theme with supporting quotes could enhance the visual presentation of the findings, making the results more accessible to readers.

## 5. Discussion
- The authors could further expand on how their findings might influence specific public health or educational policies, especially in low-resource settings, and suggest avenues for future research

## 6. Conclusion
- A brief statement highlighting the potential for community-based mental health programs tailored to the PPR context would provide a forward-looking end to the paper, emphasizing actionable insights that policymakers and practitioners can take from this research.
