# Supplementary material for: Noteworthy trends in maladaptive coping strategies and hindrances to help-seeking behaviour among adolescents living in Malaysia’s People’s Housing Project (PPR) during the COVID-19 pandemic: A qualitative study
Source: PLoS One. 2025 Mar 21;20(3):e0318381. doi: 10.1371/journal.pone.0318381 (PMC11927882; doi:10.1371/journal.pone.0318381)
Supplement: Attachment — Submitted filename: MHPPR FEEDBACK FINAL 21 JULY 2024.docx [file pone.0318381.s004.docx]

**Noteworthy Trends in Maladaptive Coping Strategies and Hindrances to Help-Seeking Behaviour Among Adolescents Living in Malaysia's People’s Housing Project (PPR) During the COVID-19 Pandemic: A Qualitative Study**

PONE-D-23-42611

Noteworthy Trends in Maladaptive Coping Strategies and Hindrances to Help-Seeking Behaviour Among Adolescents Living in Malaysia's People’s Housing Project (PPR) During the COVID-19 Pandemic: A Qualitative Study

PLOS ONE

Dear Dr. Harun,
Thank you for submitting your manuscript to PLOS ONE. After careful consideration, we feel that it has merit but does not fully meet PLOS ONE’s publication criteria as it currently stands. Therefore, we invite you to submit a revised version of the manuscript that addresses the points raised during the review process.

Please submit your revised manuscript by Jun 27 2024 11:59PM. If you will need more time than this to complete your revisions, please reply to this message or contact the journal office at plosone@plos.org. When you're ready to submit your revision, log on to <https://www.editorialmanager.com/pone/> and select the 'Submissions Needing Revision' folder to locate your manuscript file.

Please include the following items when submitting your revised manuscript:

· A rebuttal letter that responds to each point raised by the academic editor and reviewer(s). You should upload this letter as a separate file labeled 'Response to Reviewers'.

· A marked-up copy of your manuscript that highlights changes made to the original version. You should upload this as a separate file labeled 'Revised Manuscript with Track Changes'.

· An unmarked version of your revised paper without tracked changes. You should upload this as a separate file labeled 'Manuscript'.

If you would like to make changes to your financial disclosure, please include your updated statement in your cover letter. Guidelines for resubmitting your figure files are available below the reviewer comments at the end of this letter.

If applicable, we recommend that you deposit your laboratory protocols in [protocols.io](http://protocols.io/) to enhance the reproducibility of your results. Protocols.io assigns your protocol its own identifier (DOI) so that it can be cited independently in the future. For instructions see: <https://journals.plos.org/plosone/s/submission-guidelines#loc-laboratory-protocols>. Additionally, PLOS ONE offers an option for publishing peer-reviewed Lab Protocol articles, which describe protocols hosted on [protocols.io](http://protocols.io/). Read more information on sharing protocols at <https://plos.org/protocols?utm_medium=editorial-email&utm_source=authorletters&utm_campaign=protocols>.

We look forward to receiving your revised manuscript.

**Journal Requirements**

| No | Comments | Feedback |
| --- | --- | --- |
| **1.** | 1. When submitting your revision, we need you to address these additional requirements.  Please ensure that your manuscript meets PLOS ONE's style requirements, including those for file naming. The PLOS ONE style templates can be found at  <https://journals.plos.org/plosone/s/file?id=wjVg/PLOSOne_formatting_sample_main_body.pdf> and  <https://journals.plos.org/plosone/s/file?id=ba62/PLOSOne_formatting_sample_title_authors_affiliations.pdf> | |
| **2.** | We note that the grant information you provided in the ‘Funding Information’ and ‘Financial Disclosure’ sections do not match.  When you resubmit, please ensure that you provide the correct grant numbers for the awards you received for your study in the ‘Funding Information’ section. | MOH/2022/02 |
| **3.** | Thank you for stating the following financial disclosure:  Funding provided by UNICEF in the form of an unrestricted grant.   Please state what role the funders took in the study. If the funders had no role, please state: "The funders had no role in study design, data collection and analysis, decision to publish, or preparation of the manuscript."  If this statement is not correct you must amend it as needed.  Please include this amended Role of Funder statement in your cover letter; we will change the online submission form on your behalf. | The funders had no role in study design, data collection and analysis, decision to publish, or preparation of the manuscript." |
| **4.** | In the online submission form, you indicated that The datasets gathered and used in this study are not accessible to the public due to the inclusion of the confidential interview transcripts which necessitates the protection of participants’ anonymity. If additional data is needed, it can be requested through the corresponding author.  All PLOS journals now require all data underlying the findings described in their manuscript to be freely available to other researchers, either a. In a public repository, b. Within the manuscript itself, or c. Uploaded as supplementary information. This policy applies to all data except where public deposition would breach compliance with the protocol approved by your research ethics board. If your data cannot be made publicly available for ethical or legal reasons (e.g., public availability would compromise patient privacy), please explain your reasons on resubmission and your exemption request will be escalated for approval. | The data can be requested through National Institutes of Health – Data Repository System (NIH-DaRS) https://nihdars.nih.gov.my |
| **5.** | When completing the data availability statement of the submission form, you indicated that you will make your data available on acceptance. We strongly recommend all authors decide on a data sharing plan before acceptance, as the process can be lengthy and hold up publication timelines. Please note that, though access restrictions are acceptable now, your entire data will need to be made freely accessible if your manuscript is accepted for publication. This policy applies to all data except where public deposition would breach compliance with the protocol approved by your research ethics board. If you are unable to adhere to our open data policy, please kindly revise your statement to explain your reasoning and we will seek the editor's input on an exemption. Please be assured that, once you have provided your new statement, the assessment of your exemption will not hold up the peer review process. | The data can be requested through National Institutes of Health – Data Repository System (NIH-DaRS) https://nihdars.nih.gov.my/research/view/142/details |
| **6.** | PLOS requires an ORCID iD for the corresponding author in Editorial Manager on papers submitted after December 6th, 2016. Please ensure that you have an ORCID iD and that it is validated in Editorial Manager. To do this, go to ‘Update my Information’ (in the upper left-hand corner of the main menu), and click on the Fetch/Validate link next to the ORCID field. This will take you to the ORCID site and allow you to create a new iD or authenticate a pre-existing iD in Editorial Manager. Please see the following video for instructions on linking an ORCID iD to your Editorial Manager account: <https://www.youtube.com/watch?v=_xcclfuvtxQ> | 1. Siti Nur Farhana: https://orcid.org/0000-0003-2317-4057 2. Noorlaile Jasman   <https://orcid.org/0000-0001-9557-6130>   3. Dr Feisul Idzwan: 0000- <https://orcid.org/0003-3497-341X> 4. Norrafizah Jaafar:   <https://orcid.org/0000-0003-1133-2265>   5. Siti Nadiah Busyra: <https://orcid.org/0009-0001-5966-099X> 6. Dr Manimaran: <https://orcid.org/0000-0002-8554-9080> 7. Ponnusamy Subramaniam: https://orcid.org/000-0002-2361-4780 |
| **7.** | One of the noted authors is a group or consortium. In addition to naming the author group, please list the individual authors and affiliations within this group in the acknowledgments section of your manuscript. **Please also indicate clearly a lead author for this group along with a contact email address.** | We would like to clarify that none of the authors listed are part of a group or consortium. All authors are individual contributors with their respective affiliations clearly indicated in the manuscript.  The lead author for this group is Siti Nur Farhana binti Harun, and she can be contacted at farhana@moh.gov.my |
| **8.** | Please amend either the abstract on the online submission form (via Edit Submission) or the abstract in the manuscript so that they are identical. | Amended. |
| **9.** | Your ethics statement should only appear in the Methods section of your manuscript. If your ethics statement is written in any section besides the Methods, please move it to the Methods section and delete it from any other section. Please ensure that your ethics statement is included in your manuscript, as the ethics statement entered into the online submission form will not be published alongside your manuscript. | Amended.  **Kindly refer to line 185-187** |

**Reviewer's Responses to Questions**

**Comments to the Author**

| No | Comments | Feedback |
| --- | --- | --- |
| **1.** | Is the manuscript technically sound, and do the data support the conclusions?    The manuscript must describe a technically sound piece of scientific research with data that supports the conclusions. Experiments must have been conducted rigorously, with appropriate controls, replication, and sample sizes. The conclusions must be drawn appropriately based on the data presented.  Reviewer #1: Yes  Reviewer #2: Partly | The manuscript has been improved to make sure the conclusion reflects the data presented. |
| **2.** | Has the statistical analysis been performed appropriately and rigorously?  Reviewer #1: N/A  Reviewer #2: Yes | Statistical analysis been performed appropriately and rigorously. |
| **3.** | Have the authors made all data underlying the findings in their manuscript fully available?    The PLOS Data policy requires authors to make all data underlying the findings described in their manuscript fully available without restriction, with rare exception (please refer to the Data Availability Statement in the manuscript PDF file). The data should be provided as part of the manuscript or its supporting information, or deposited to a public repository. For example, in addition to summary statistics, the data points behind means, medians and variance measures should be available. If there are restrictions on publicly sharing data—e.g. participant privacy or use of data from a third party—those must be specified.  Reviewer #1: Yes  Reviewer #2: No | In the revised manuscript, we have included the data availability statement.  The data can be requested through National Institutes of Health – Data Repository System (NIH-DaRS) https://nihdars.nih.gov.my/ |
| **4.** | 4. Is the manuscript presented in an intelligible fashion and written in standard English?    PLOS ONE does not copyedit accepted manuscripts, so the language in submitted articles must be clear, correct, and unambiguous. Any typographical or grammatical errors should be corrected at revision, so please note any specific errors here.  Reviewer #1: Yes  Reviewer #2: Yes | Thank you for the positive feedback. |

**Reviewer 1**

| No | Comments | Feedback |
| --- | --- | --- |
|  | Reviewer #1: In this paper the authors conducted a qualitative study to examine coping strategies and barriers to help-seeking among adolescents living in people’s housing projects during the COVID-19 pandemic.  The vulnerability of the study population adds to the importance of this paper, and the emphasis on promoting adaptive coping strategies among this subgroup is a well-received take-home message.  A strength of the present study is its rigorous standard for study design and procedures, while the manuscript itself is clear and generally well-written.    Below are my specific comments that are aimed at strengthening the paper: | |
| **1.** | In the Introduction, the authors can provide contextual information on PPR and Klang Valley to help readers who are unfamiliar with the context appreciate why the study population are vulnerable and merit scientific importance, and better appreciate the paper’s contribution. | Thank you for highlighting this important point, we have included new text as per reviewer’s suggestion.  **Kindly refer to line 76-81** |
| **2.** | p.8, under the Externalization subheading: Describing participants as resorting to destructive behaviours such as hitting their mother sounded like it was done on purpose, which would be misleading in my opinion as R9’s quoted response seemed to suggest otherwise.  The authors may wish to clarify and/or revise accordingly. | Thank you for your valuable feedback. We appreciate your observation regarding the description of participants' behaviours under the Externalization subheading. We understand the concern that the wording might imply intentional harm, which is not aligned with the participants' experiences as described.  In response, we have revised the section to more accurately reflect the participants' lack of control during these incidents of destructive behaviour. We aim to clarify that these actions were not intentional but rather a result of overwhelming emotional distress.  This revision clarifies that the participants' behaviours were not deliberate acts of harm but rather impulsive reactions to intense emotions. We hope this addresses the concern and provides a clearer understanding of the participants' experiences.  **Kindly refer to line 284-291** |
| **3.** | p.13, under the Discussion section: The authors may need to expound further on the sentence “This trend was not only observed among the participants in this study but also occurred elsewhere.” The specific word “elsewhere” is vague, and here the discussion may be enriched with specific examples and/or citations to explicitly engage with the literature – either for similarly vulnerable adolescents within the Asian Pacific context or other sociocultural contexts. | Thank you for your valuable feedback.  **Kindly refer to line 76-81** |
| **4.** | Minor comments:  - The second sentence under the Methods section is a repeat of the first.  - There seems to be a typo in the second sentence of Strengths and Limitations: “we were able to capture”?  - If relevant, the authors may consider adding interview guidelines and/or structure as supplementary materials. | Thank you for your valuable feedback.   - The repetitive sentence (page 5) has been deleted. - The typo has been rectified. - interview guidelines has been added as supplementary materials. |

**Reviewer 2**

| No | Comments | Feedback |
| --- | --- | --- |
|  | Introduction  1. The introduction does not clearly establish the novelty of the study. It would benefit from a more detailed explanation of how this study fills a specific gap in the existing literature on adolescents' coping strategies in Malaysia or similar settings. | Thank you for your feedback. The novelty of this research lies in its focus on promoting adaptive coping strategies among underprivileged adolescents in urban poor settings in Malaysia. To the best of our knowledge, there are limited studies on coping strategies in this specific population especially during COVID-19 pandemic which highlights the importance and uniqueness of our study. We have now emphasized this aspect more clearly in the introduction.  **Kindly refer to line 138-145** |
|  | 2.The rationale for focusing on adolescents in Malaysia's People’s Housing Project (PPR) during the COVID-19 pandemic is inadequately explored. There's a need for more specific data or previous studies that highlight unique issues faced by this demographic that warrant this research | Thank you for highlighting this point, we have revised the manuscript based on reviewer’s comment.  **Kindly refer to line 83-89** |
|  | Methods  3. The purposive sampling method is described but lacks a detailed justification for why this method ensures a representative sample of the adolescent population in the PPRs, potentially biasing the results. | Thank you for your valuable feedback. This is a qualitative study and is not intended to represent the entire adolescent population in the PPR, but to explore in depth each participant’s unique experience with coping strategies and hindrances to help-seeking behaviour among adolescents living in Malaysia's People’s Housing Project (PPR) during the COVID-19 pandemic.  However, the homogeneity of the sample population, in terms of shared socio-economic conditions and experiences within the PPRs, helps ensure that the insights gathered are relevant and reflective of the specific challenges faced by this group. By focusing on this particular setting, we aim to provide a detailed understanding of the coping mechanisms and barriers to help-seeking in a context that is often underrepresented in research. This approach allows us to uncover detailed, context-specific information that can inform targeted interventions and support services for these vulnerable adolescents.  The limitation of the qualitative research highlighted in the discussion section. |
|  | 4. The article mentions a semi-structured interview guide based on specific theories but fails to provide enough detail about the questions asked. This omission limits the replicability of the study. | The Transactional Theory of Stress and Coping Model informed the structure of our interview guide, particularly around primary and secondary appraisals of stress, as well as the use of problem-focused and emotion-focused coping strategies. Primary appraisals involved assessing whether situations were perceived as positive, threatening, or challenging. Secondary appraisals considered the availability of social and psychological resources for coping.  **Kindly refer to line 176-181**  While the overview of the theoretical framework guides our study, we recognize the importance of methodological transparency. In response to your feedback, a detailed outline of the interview questions is included in the supplement to enhance the replicability of our study. |
|  | 5. While the study obtained ethics approval and parental consent, it does not discuss how it addressed potential emotional distress caused to participants discussing their coping strategies and mental health, which is crucial given the sensitive nature of the subject. | Thank you for your comment, we have highlighted the Intervention for Participants with Psychological Distress.  **Kindly refer to line 159-172** |
|  | Results  6. The results section provides an aggregate view but lacks a detailed demographic breakdown that could reveal important patterns or differences in coping strategies among different age groups or between genders. | Thank you for pointing this out. We have included detailed information in the revised manuscript as follows; “From the 37 PPRs, a total of 1578 eligible participants’ ages ranged from 10 to 17 years (M = 13.52, SD = ±2.193) with females 50.5% and males 49.5% old completed the questionnaire. Based on the total number of eligible participants, 194 adolescents were identified as having psychological distress based on the screening.”  **Kindly refer to line 227-230** |
|  | 7. The claim of data saturation is made after 42 interviews, but there is no explanation of **how this was determined** or why additional interviews were conducted if saturation was achieved. | For this study, saturation was achieved after 42 interviews, where no new information emerged across identified domains. Five additional interviews were conducted as a confirmatory measure reaffirming that saturation had indeed been reached. In many studies, even after claiming saturation, additional data is collected to verify this claim (1)  (Saunders B, Sim J, Kingstone T, Baker S, Waterfield J, Bartlam B, et al. Saturation in qualitative research: exploring its conceptualization and operationalization. Qual Quant. 2018;52(4):1893-907.)  **Kindly refer to line 231-235** |
|  | Discussion   8. The discussion does not adequately compare the study’s findings with existing research. For instance, how do the maladaptive strategies identified align or differ from those found in other cultural or socioeconomic settings? | Thank you for your valuable feedback.  We have added following paragraph in our discussion.  “As reported in this study, globally it was well established that COVID-19 pandemic increased anxiety and depression among adolescents (20) (21). Our current findings on maladaptive coping strategies in line with many previous literatures. A year follow-up study with 153 students in Italy after COVID-19 pandemic shows increase in maladaptive behaviours such as unprotected sex, self-harm ideation, self-harm behaviours, binge eating and aggressive behaviours (22). Another study with 2280 Slovak youth chooses extreme maladaptive coping strategies which are deliberate self-harm behaviours and this is prevalent among female adolescents as reported in our study (23). Furthermore, a qualitative finding indicates significant number of adolescents have tendency towards adopting maladaptive coping strategies and refuse to seek professional support (24) as indicated in current study”  **Kindly refer to line 430-439** |
|  | 9. The discussion makes broad generalizations about the prevalence of psychological distress and coping strategies among adolescents without considering the limitations inherent in qualitative research, such as the lack of generalizability. 10. The discussion does not address the methodological limitations, such as potential biases in self-reporting and the impact of the interviewer’s presence on participants' responses. | Response 9 & 10:  We have added following paragraph in our discussion.  “Although this study explores coping strategies and help-seeking behaviours among adolescents using in depth qualitative interview, they still lacked in generalizability. Methodologically, qualitative studies itself don’t provide objective outcomes which can further limits the generalizability of current findings (50, 51). Qualitative studies offer subjective insights based on individual experiences and because this method do not yield quantifiable results, it can be challenging to apply the findings broadly to other contexts or populations.  Secondly, the presence of the researcher and counsellor during the may affect participants’ responses which may leading to biased results. Thirdly, self-reporting data can provide rich information, participants’ perspectives and opinion on specific issues but biases may occur due to conditions such as selective recalls, social preferability (52) and false memories. Thus, adolescences in this study may cause biases in recalling and reporting their experiences during COVID-19 pandemic”.  **Kindly refer to line 571-584** |
|  | Conclusion  11. The conclusion emphasizes the need for interventions without a critical examination of the practical challenges or potential barriers in implementing such programs in the PPR context. | We have included new text on this issue “Mental health literacy programs can increase help-seeking willingness and adaptive coping through education and stigma reduction; however, the local community must be engaged to understand the local issues and challenges in implementing these programs”  **Kindly refer to line 618-622** |
|  | 12. The paper does not suggest areas for future research or how subsequent studies could overcome the limitations identified in this study. | We have included some new text to provide a selection of possible areas of future research, while linking to the issues raised in item #13 as well.  **Kindly refer to line 607-616** |
|  | 13. While the study aims to inform policy, it provides vague recommendations for policymakers without concrete steps or consideration of policy implementation challenges. | **Kindly refer to line 618-626** |
