# Supplementary material for: Noteworthy trends in maladaptive coping strategies and hindrances to help-seeking behaviour among adolescents living in Malaysia’s People’s Housing Project (PPR) during the COVID-19 pandemic: A qualitative study
Source: PLoS One. 2025 Mar 21;20(3):e0318381. doi: 10.1371/journal.pone.0318381 (PMC11927882; doi:10.1371/journal.pone.0318381)
Supplement: Attachment — Submitted filename: Response to Reviewers.docx [file pone.0318381.s005.docx]

**Noteworthy Trends in Maladaptive Coping Strategies and Hindrances to Help-Seeking Behaviour Among Adolescents Living in Malaysia's People’s Housing Project (PPR) During the COVID-19 Pandemic: A Qualitative Study**

PONE-D-23-42611R1

Noteworthy trends in maladaptive coping strategies and hindrances to help-seeking behaviour among adolescents living in Malaysia's People’s Housing Project (PPR) during the COVID-19 pandemic: a qualitative study

PLOS ONE

Dear Dr. Subramaniam,

Thank you for submitting your manuscript to PLOS ONE. After careful consideration, we feel that it has merit but does not fully meet PLOS ONE’s publication criteria as it currently stands. Therefore, we invite you to submit a revised version of the manuscript that addresses the points raised during the review process.

Please submit your revised manuscript by Dec 29 2024 11:59PM. If you will need more time than this to complete your revisions, please reply to this message or contact the journal office at [plosone@plos.org](mailto:plosone@plos.org). When you're ready to submit your revision, log on to <https://www.editorialmanager.com/pone/> and select the 'Submissions Needing Revision' folder to locate your manuscript file.

Please include the following items when submitting your revised manuscript:

- **A rebuttal letter that responds to each point raised by the academic editor and reviewer(s). You should upload this letter as a separate file labeled 'Response to Reviewers'.**
- **A marked-up copy of your manuscript that highlights changes made to the original version. You should upload this as a separate file labeled 'Revised Manuscript with Track Changes'.**
- **An unmarked version of your revised paper without tracked changes. You should upload this as a separate file labeled 'Manuscript'.**

If you would like to make changes to your financial disclosure, please include your updated statement in your cover letter. Guidelines for resubmitting your figure files are available below the reviewer comments at the end of this letter.

If applicable, we recommend that you deposit your laboratory protocols in [protocols.io](http://protocols.io/) to enhance the reproducibility of your results. Protocols.io assigns your protocol its own identifier (DOI) so that it can be cited independently in the future. For instructions see: [https://journals.plos.org/plosone/s/submission-guidelines#loc-laboratory-protocols](https://track.editorialmanager.com/CL0/https:%2F%2Fjournals.plos.org%2Fplosone%2Fs%2Fsubmission-guidelines%23loc-laboratory-protocols/1/010f01932bed44a8-68b28717-c372-4879-b731-b84bcf29e6bf-000000/X3ChRyZvGwcdeODdbNkgmXzPDQKdqv_F8-Y5jwE5qj8=185). Additionally, PLOS ONE offers an option for publishing peer-reviewed Lab Protocol articles, which describe protocols hosted on [protocols.io](http://protocols.io/). Read more information on sharing protocols at [https://plos.org/protocols?utm_medium=editorial-email&utm_source=authorletters&utm_campaign=protocols](https://track.editorialmanager.com/CL0/https:%2F%2Fplos.org%2Fprotocols%3Futm_medium=editorial-email%26utm_source=authorletters%26utm_campaign=protocols/1/010f01932bed44a8-68b28717-c372-4879-b731-b84bcf29e6bf-000000/KQ9zeJWMbyiSMpq6_n-rGl7LLJWdJ2FiKef9lpX-rBg=185).

We look forward to receiving your revised manuscript.

**Response to Reviewers**

**Reviewer 3**

| No | Comments | Feedback |
| --- | --- | --- |
| **1.** | 1.Abstract 1. Clarity and Structure: The abstract could benefit from clearer wording, particularly in describing the research question and the study's significance. Opening with a concise statement on the specific challenges faced by adolescents in Malaysia’s People’s Housing Project (PPR) communities due to the COVID-19 pandemic would improve focus. | Thank you for your valuable feedback. We appreciate your observation regarding the relevance of this study to the PPR community. In the abstract we added below sentences to highlight the relevance of the research question as per suggestion.  ***Line 29-31:*** *“Adolescents residing in PPR communities are among the most vulnerable groups of young people in urban areas, given their pre-existing conditions of vulnerability, face even greater challenges due to the pandemic”*  ***Line 33-36:*** *“This study is to explore the coping strategies and barriers to help-seeking behaviour among adolescents living in Malaysia's People's Housing Project (PPR) communities, focusing on the unique mental health challenges exacerbated by the COVID-19 pandemic.”* |
|  | 2. Relevance of the Research Question: Adding a sentence on why this study is particularly relevant to PPR communities in Malaysia would strengthen the justification and make the abstract more compelling. | Thank you for pointing out this important point. We have highlighted this sentence at the revised abstract in the latest manuscript;  ***Line 36-38:*** *"Given the socio-economic vulnerabilities and the heightened mental health challenges during the pandemic, this study provides critical insights into how adolescents in PPR communities navigate psychological distress and access mental health support."*  This addition strengthens the justification by connecting the study's importance to the specific context of PPR communities and their unique struggles during the pandemic. |
|  | 3. Methods: Consider adding a phrase on the sample size (e.g., number of participants) and the specific methods used (e.g., semi-structured interviews), as this provides readers with a clearer understanding of the study's scope. | Thank you for your valuable feedback.  We have highlighted this in the revised manuscript;  ***Line 41-43:*** *“This qualitative study used a phenomenological research design and was conducted from January to December 2022 involved 47 adolescents aged 10 to 17 years old from 37 People’s Housing Project in the Klang Valley.”*  ***Line 47-48:*** *“Data were collected using a semi-structured interview guide to conduct the in-depth interviews (IDI).”* |
|  | 4. Results :To improve clarity, specify some of the maladaptive coping strategies and barriers identified (e.g., avoidance, lack of trust in mental health services). Including one or two specific examples would make the findings more tangible for the reader. | Thank you for your valuable feedback.  We have highlighted this suggestion in the revised manuscript;  ***Line 57-61:*** *Among them, 47 agreed to participate in in-depth interviews (IDIs), which revealed adolescents utilized maladaptive coping strategies, such as avoidance (cognitive distancing, externalization, and internalization), self-harm, vaping, and smoking to deal with stressors related to COVID-19. As for hindrances to help-seeking, three themes were identified such as lack of trust, perceived ineffectiveness of support, and personality.* |
|  | 5. Conclusions and Implications: Strengthen the conclusion by mentioning potential implications for policy or practice. For example, a brief statement about how this research could inform mental health interventions tailored to disadvantaged communities would make the abstract more impactful. | Thank you for pointing this out. We have included the statement about how this research could inform mental health interventions tailored to disadvantaged communities  ***Line 67-75:*** *These findings emphasize the need for targeted mental health interventions and support systems tailored to vulnerable communities. These interventions could inform policies aimed at strengthening mental health services, fostering adaptive coping strategies and promoting help-seeking behaviours among adolescents in socio-economically challenged setting.* |
| **2.** | **2. Introduction** • To strengthen the relevance, consider providing a brief overview of the unique characteristics of the PPR communities (e.g., socio-economic constraints, limited access to mental health resources). This would contextualize the challenges faced by adolescents in these settings and reinforce the importance of the study. | ***Line 96-103:*** *The dense, crowded and low-resource setting predominantly experienced in PPR communities, often means that children based in these living environments are more susceptible to socio-economic deprivations, and are at higher risk of developing mental health conditions.* |
|  | • While the authors have referenced international studies on adolescent coping and help-seeking, the literature review could benefit from further discussion of any regional or cultural factors specific to Southeast Asia or Malaysia, which might influence coping strategies and help-seeking behaviors. This would provide additional context for the study’s relevance | Thank you for your valuable feedback.  We have highlighted this suggestion in the revised manuscript.  **Kindly please refer to line 148-153** |
|  | • Consider explicitly stating the research question or hypothesis at the end of the introduction. This would give readers a clear understanding of the study’s focus and objectives. | **Kindly please refer to line 157 to 160** |
| **3.** | 3. Methods • A more detailed explanation of the coding and analysis process would improve transparency. Specifically, it would be beneficial to describe how coding was conducted (e.g., use of software, independent coding by multiple researchers) and how disagreements were resolved to ensure inter-coder reliability. | **Kindly please refer to line 245-262** |
|  | • Additionally, mentioning whether the researchers used any **form of reflexivity to minimize bias** would strengthen the methodological robustness. | Thank you for pointing this out. We have included the statement about how used reflexivity to minimize researchers’ biases;  **Kindly please refer to line 209-213**  *In this study, reflexivity was used as a strategy to reduce researcher bias by reflecting on how personal perspectives, assumptions and beliefs could influence data analysis and interpretation. Debriefing and research team member checking were employed as tools to critically examine the researcher's role and potential biases throughout the research process.* |
| **4.** | 4. Results • Including some quantitative data, such as the proportion of participants endorsing each coping strategy or barrier, would provide additional context and help quantify the prevalence of specific behaviors. | Thank you for the suggestion regarding the inclusion of quantitative data to provide additional context. However, this study is rooted in a qualitative methodology, which is designed to explore in-depth,and rich insights rather than quantify behaviours or experiences. As such, quantifying the proportion of participants endorsing specific coping strategies or barriers would not align with the principles of this qualitative approach.  Instead, we have prioritized thematic analysis to highlight the recurring patterns and variations in coping strategies and barriers among participants. This approach allows us to provide detailed and contextualized understandings of the lived experiences of adolescents in PPR communities. While we do not present numerical prevalence, we ensure transparency and rigor by supporting our findings with representative quotes and robust thematic evidence. |
|  | • Furthermore, a summary table outlining each theme with supporting quotes could enhance the visual presentation of the findings, making the results more accessible to readers. | Thank you for your suggestion. Kindly refer to **table 2** |
| **5** | 5. Discussion • The authors could further expand on how their findings might influence specific public health or educational policies, especially in low-resource settings, and suggest avenues for future research | Thank you for your valuable feedback.  We have highlighted this suggestion in the revised manuscript;  **Kindly refer to line 612 to 619.**  **As for future research, kindly refer to line 658 to 667.**  *“Future research on adolescents' mental health during and after the COVID-19 pandemic should consider several directions to overcome limitations identified in current studies. Longitudinal studies are essential to track changes in coping strategies and help-seeking behaviours over time, offering insights into long-term effects. Intervention studies tailored to vulnerable communities, like psycho-education and resilience training, can test effectiveness in promoting adaptive coping strategies. Comparative studies between adolescents in different community types such as urban, rural and affluent settings can highlight unique challenges, aiding targeted intervention design. Community-Based Participatory Research (CBPR) involving adolescents, parents, educators, and local organizations ensures culturally relevant interventions and builds trust.”* |
| **6** | 6. Conclusion • A brief statement highlighting the potential for community-based mental health programs tailored to the PPR context would provide a forward-looking end to the paper, emphasizing actionable insights that policymakers and practitioners can take from this research. | **Kindly refer to line 692 to 699**  *By highlighting the coping strategies and barriers to help-seeking behaviours, the findings provide valuable insights into how adolescents in low-resource settings navigate psychological distress. These findings emphasize the critical need for community-based mental health programs tailored to the PPR context. Such initiatives could focus on fostering adaptive coping strategies, reducing stigma around mental health, and improving access to support services. Policymakers and practitioners can leverage these insights to develop targeted interventions that promote mental health and resilience among vulnerable adolescents, ensuring their well-being in both times of crisis and beyond.* |
