# Supplementary material for: Noteworthy trends in maladaptive coping strategies and hindrances to help-seeking behaviour among adolescents living in Malaysia’s People’s Housing Project (PPR) during the COVID-19 pandemic: A qualitative study
Source: PLoS One. 2025 Mar 21;20(3):e0318381. doi: 10.1371/journal.pone.0318381 (PMC11927882; doi:10.1371/journal.pone.0318381)
Supplement: Attachment — Submitted filename: Response_to_Reviewers_auresp_3.docx [file pone.0318381.s006.docx]

PONE-D-23-42611R2

**Noteworthy trends in maladaptive coping strategies and hindrances to help-seeking behaviour among adolescents living in Malaysia's People’s Housing Project (PPR) during the COVID-19 pandemic: a qualitative study**

PLOS ONE

Dear Dr. Subramaniam,

Thank you for submitting your manuscript to PLOS ONE. After careful consideration, we feel that it has merit but does not fully meet PLOS ONE’s publication criteria as it currently stands. Therefore, we invite you to submit a revised version of the manuscript that addresses the points raised during the review process.

**These are revisions from me the Academic Editor. I** see that you have answered all the queries of the last Reviewer, however, the underlisted remain before the paper can be accepted. They are editing tasks and I encourage you to work on them.

Thank you.

REVISIONS AUTHOR NEEDS TO WORK ON—MOSTLY  EDITING

FOR THE REVISED SECTIONS

P. 2, line 68: **This findings** study emphasize the need for

p. 5, Line 144: Additionally, in the context of Asian nations and low and middle income countries, 144 cultural factors such as stigma surrounding mental health **issue (**16), familial support systems 145 (17) and awareness of mental health (18) may further shape adolescents’ coping strategies 146 and help-seeking behaviours. Addressing these cultural significance is critical to 147 understanding and effectively intervening in mental health challenges faces by this 148 population. 149 150 Hence, th

P. 8, line 245: coding framework. Within these discussions, **the** research team also identified and incorporated (YOU OMITTED ‘THE’)

FOR THE ORIGINAL SECTIONS THAT WERE NOT REVISED

P. 22, line 612: provide objective outcomes which can further **limits** the generalizability of current findings (SHOULD BE ‘LIMIT’)

P. 22, line 617: Secondly, the presence of the researcher and counsellor during the **(OMISSION HERE)** may affect

P. 22, line 618: participants’ responses which **may leading** to biased results (SOMETHING IS NOT RIGHT WITH ‘MAY LEADING’….

Please include the following items when submitting your revised manuscript:

- A rebuttal letter that responds to each point raised by the academic editor and reviewer(s). You should upload this letter as a separate file labeled 'Response to Reviewers'.
- A marked-up copy of your manuscript that highlights changes made to the original version. You should upload this as a separate file labeled 'Revised Manuscript with Track Changes'.
- An unmarked version of your revised paper without tracked changes. You should upload this as a separate file labeled 'Manuscript'.

If you would like to make changes to your financial disclosure, please include your updated statement in your cover letter. Guidelines for resubmitting your figure files are available below the reviewer comments at the end of this letter.

If applicable, we recommend that you deposit your laboratory protocols in [protocols.io](http://protocols.io/) to enhance the reproducibility of your results. Protocols.io assigns your protocol its own identifier (DOI) so that it can be cited independently in the future. For instructions see: [https://journals.plos.org/plosone/s/submission-guidelines#loc-laboratory-protocols](https://track.editorialmanager.com/CL0/https:%2F%2Fjournals.plos.org%2Fplosone%2Fs%2Fsubmission-guidelines%23loc-laboratory-protocols/1/010f01942afa21a5-21e17264-0dda-49c9-a86f-4b2d27be0fce-000000/PiAr2FyrKnBaOhrzMdITAuSs_z5jA2ky096eFMNu4rM=191). Additionally, PLOS ONE offers an option for publishing peer-reviewed Lab Protocol articles, which describe protocols hosted on [protocols.io](http://protocols.io/). Read more information on sharing protocols at [https://plos.org/protocols?utm_medium=editorial-email&utm_source=authorletters&utm_campaign=protocols](https://track.editorialmanager.com/CL0/https:%2F%2Fplos.org%2Fprotocols%3Futm_medium=editorial-email%26utm_source=authorletters%26utm_campaign=protocols/1/010f01942afa21a5-21e17264-0dda-49c9-a86f-4b2d27be0fce-000000/__PP4AbTQebL73VgkWeqsCGsp5_wp9YJtAMxnPD_XbQ=191).

We look forward to receiving your revised manuscript.

Kind regards,

Adobea Yaa Owusu, MA, PhD, MPH
Academic Editor
PLOS ONE

Journal Requirements:

Please review your reference list to ensure that it is complete and correct. If you have cited papers that have been retracted, please include the rationale for doing so in the manuscript text, or remove these references and replace them with relevant current references. Any changes to the reference list should be mentioned in the rebuttal letter that accompanies your revised manuscript. If you need to cite a retracted article, indicate the article’s retracted status in the References list and also include a citation and full reference for the retraction notice.

**Response to Academic Editor**

| No | Comments | Feedback |
| --- | --- | --- |
| **1.** | P. 2, line 68: **This findings** study emphasize the need for | Thank you for your valuable feedback.  ***Line 67: These*** *findings* |
|  | p. 5, Line 144: Additionally, in the context of Asian nations and low and middle income countries, 144 cultural factors such as stigma surrounding mental health **issue (**16), familial support systems 145 (17) and awareness of mental health (18) may further shape adolescents’ coping strategies 146 and help-seeking behaviours. Addressing these cultural significance is critical to 147 understanding and effectively intervening in mental health challenges faces by this 148 population. 149 150 Hence, th | ***Line 161:*** *Health* ***issues*** |
|  | P. 8, line 245: coding framework. Within these discussions, **the** research team also identified and incorporated (YOU OMITTED ‘THE’) | ***Line 267: The*** *research team* |
|  | P. 22, line 612: provide objective outcomes which can further **limits** the generalizability of current findings (SHOULD BE ‘LIMIT’) | ***Line 749:*** ***limit*** *the generalizability* |
|  | P. 22, line 617: Secondly, the presence of the researcher and counsellor during the **(OMISSION HERE)** may affect | ***Line 754:*** *Secondly, the presence of the researcher and counsellor during the* ***IDIs*** *may affect* |
|  | P. 22, line 618: participants’ responses which **may leading** to biased results (SOMETHING IS NOT RIGHT WITH ‘MAY LEADING’…. | ***Line 754- 755:***  *Secondly, the presence of the researcher and counsellor during the IDIs may affect participants’ responses.*  *which* ***may leading*** *to biased results (OMITTED)* |
|  | **We have addressed all the grammatical issues raised by the reviewer - additionally we detected and corrected several others - as shown by track changes in the revised version of the manuscript.** | |
